# An Overview of the Impact of the Menstrual Cycle on Nutrient Metabolism: An Integrative Perspective

**DOI:** 10.3390/nu18071063

**Published:** 2026-03-26

**Authors:** Cielo García-Montero, Patricia de Castro-Martínez, Diego Liviu Boaru, Miguel A. Ortega, Óscar Fraile-Martínez

**Affiliations:** 1 Department of Medicine and Medical Specialities, Faculty of Medicine and Health Sciences, University of Alcala, 28801 Alcala de Henares, Spain; patriciadecastro1999@gmail.com (P.d.C.-M.); diegoboaru@hotmail.com (D.L.B.); miguel.angel.ortega92@gmail.com (M.A.O.); oscarfra.7@gmail.com (Ó.F.-M.); 2 Ramón y Cajal Institute of Sanitary Research (IRYCIS), 28034 Madrid, Spain

**Keywords:** menstrual cycle, nutrient metabolism, ovarian hormones, metabolic regulation, energy metabolism, micronutrient metabolism, substrate utilization, metabolic flexibility, precision nutrition

## Abstract

The menstrual cycle represents a dynamic infradian rhythm characterized by coordinated fluctuations in ovarian steroids that extend beyond reproductive function and influence systemic metabolism. This narrative review synthesizes current evidence on how menstrual cycle phase modulates energy balance, macronutrient metabolism, micronutrient handling, and responses to dietary bioactive compounds. Across phases, small-to-moderate but consistent differences emerge in energy intake, resting energy expenditure, substrate utilization, and protein turnover, with a tendency toward increased energy intake and lipid oxidation during the mid-luteal phase compared with the early follicular and peri-ovulatory phases. Emerging metabolomics data further reveal coordinated cyclical variation in amino acids, B vitamins, and lipid species, suggesting temporally sensitive windows in which low energy availability or micronutrient insufficiency may more readily impair performance, recovery, or symptom burden. Importantly, menstrual cycle-related metabolic variability reflects not only estradiol and progesterone oscillations but also integrated adaptations across the hypothalamic–pituitary–adrenal axis, autonomic nervous system, immune signaling, and gut microbiota. These interconnected systems contribute to inter- and intra-individual heterogeneity in metabolic phenotype. From a clinical and applied perspective, the evidence supports “cycle-aware” but non-dogmatic nutritional strategies, particularly in contexts of metabolic dysfunction, high training loads, or reproductive disorders. Future research should systematically verify cycle phase, incorporate multi-system biomarkers, and adopt sex-specific analytical frameworks to improve translational relevance. Recognizing the menstrual cycle as a biologically meaningful metabolic variable may enhance precision nutrition, exercise prescription, and metabolic risk assessment in women.

## 1. Introduction

Female nutrient and energy metabolism are governed by a uniquely dynamic endocrine landscape that operates across both cyclical (menstrual) and lifespan-dependent timescales [1,2]. Unlike the relatively stable hormonal environment in men, women experience cyclical variations in 17β-estradiol (E2) and progesterone (P4) that directly influence key metabolic processes, including glucose homeostasis, lipid oxidation, amino acid turnover, and mitochondrial bioenergetics [3,4,5]. These hormonal effects are mediated through both genomic and non-genomic mechanisms, regulating enzyme activity, substrate transport, and mitochondrial function [6]. Despite this, female-specific metabolic physiology has historically been underrepresented in research, and current evidence remains fragmented across molecular, clinical, and applied domains, limiting the ability to generate an integrated understanding of nutrient metabolism across the menstrual cycle.

The hypothalamic–pituitary–gonadal (HPG) axis, commonly referred to as the hypothalamic–pituitary ovarian (HPO) axis in women-, underlies these cyclical hormonal changes by coordinating the production of E2 and P4 across the menstrual cycle. Pulsatile gonadotropin-releasing hormone (GnRH) secretion from the hypothalamus drives follicle-stimulating hormone (FSH) and luteinizing hormone (LH) release from the pituitary, thereby regulating ovarian steroidogenesis (estrogen and progesterone) [7]. The menstrual cycle synchronizes ovarian and endometrial processes to generate distinct metabolic milieus across follicular, ovulatory, and luteal phases [2]. During the early follicular phase, low concentrations of E2 and P4 are associated with a metabolic profile characterized by relatively higher carbohydrate reliance. In contrast, the late follicular and peri-ovulatory phases, marked by rising E2 levels, are associated with increased lipid oxidation and improved metabolic efficiency. The luteal phase, characterized by elevated P4 and moderate E2 levels, induces a distinct metabolic state involving shifts in substrate utilization, increased energy expenditure, and changes in insulin sensitivity [8].

Importantly, these phase-dependent metabolic responses do not occur in isolation but are modulated by interacting physiological systems, including inflammation, the gut microbiota, circadian rhythms, physical activity, and behavioral factors [9,10,11,12,13,14]. These interactions shape the metabolic context in which hormonal signaling operates, contributing to interindividual variability in nutrient metabolism across the menstrual cycle. Recent work has increasingly emphasized the role of these integrative systems—including energy availability, microbiota-derived metabolites, and neuroendocrine stress responses—in modulating or even overriding hormonal effects [15,16,17,18,19], highlighting the need for a systems-level perspective.

However, while several studies have examined specific aspects of female metabolism in isolation, few have comprehensively integrated molecular mechanisms with whole-body metabolism and real-world modifiers across the menstrual cycle. This gap limits translational applicability, particularly for fields such as sports nutrition, metabolic health, and personalized medicine.

Accordingly, this review focuses on the mechanistic regulation of nutrient metabolism across the menstrual cycle. By integrating molecular, physiological, and applied evidence, we aim to clarify how cyclical variations in E2 and P4 influence substrate utilization, metabolic flexibility, and mitochondrial function. This approach seeks to provide a more precise framework for interpreting metabolic data in women and to inform cycle-aware nutritional and exercise strategies, while acknowledging the influence of individual variability and contextual factors.

## 2. Methodology

This manuscript was designed as a narrative and integrative review rather than a systematic review, with the aim of synthesizing and critically interpreting the most relevant and mechanistically informative evidence on menstrual cycle physiology and nutrient metabolism.

**Figure 1:**
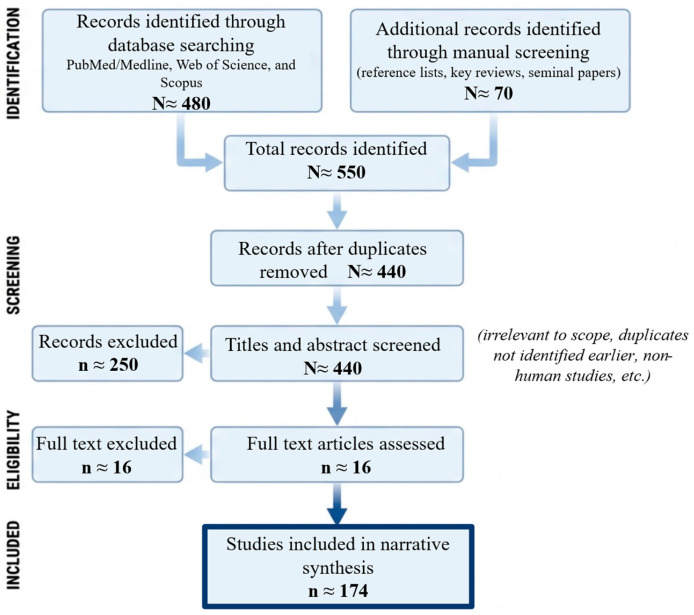
**Literature search and study selection process**. PRISMA-inspired flowchart illustrating the structured literature search and selection strategy used in this narrative review. Records were identified through database searching (PubMed/MEDLINE, Web of Science, and Scopus) and complemented by manual screening of reference lists and key reviews. Following duplicate removal, titles and abstracts were screened for relevance, and full-text articles were assessed based on predefined thematic and methodological criteria. Studies were included if they provided mechanistic, physiological, or applied insights into menstrual cycle-related variations in nutrient metabolism. Due to the narrative and integrative design of this review, no formal systematic review procedures (e.g., risk-of-bias assessment or PRISMA registration) were applied. Accordingly, the reported numbers should be interpreted as approximate and are provided to enhance transparency rather than to represent an exhaustive or reproducible systematic search.

To enhance transparency, a structured literature search was conducted across major biomedical databases (PubMed/MEDLINE, Web of Science, and Scopus), complemented by manual screening of reference lists and key reviews. The search covered publications available up to 28 February 2026 and combined controlled vocabulary and free-text terms related to ovarian hormones, nutrient metabolism, metabolic regulation, inflammation, microbiota, and behavioral factors. The overall selection process is summarized in Figure 1.

Records identified through database searching and manual screening were first evaluated based on titles and abstracts, followed by full-text assessment of studies considered relevant. Inclusion was based on direct relevance to menstrual cycle-related metabolic variability and contribution to mechanistic, physiological, or applied understanding. Given the interdisciplinary nature of the topic, studies were selected across predefined thematic domains, including hormonal regulation, macronutrient and micronutrient metabolism, mitochondrial function, exercise metabolism, inflammation, microbiota, and behavioral factors.

Priority was given to peer-reviewed human studies (including controlled and longitudinal designs), meta-analyses, and high-quality reviews. When human evidence was limited, well-designed experimental or translational studies were included to support mechanistic interpretation. Studies were generally excluded if they lacked clear characterization of menstrual cycle phase, were non-peer-reviewed, or provided insufficient methodological detail.

Particular attention was paid to methodological quality indicators, including adequacy of cycle phase verification (e.g., hormonal confirmation), study design robustness, and participant characteristics. Preference was given to recent literature (last 10–15 years), while seminal studies were included when necessary for conceptual context.

Due to its narrative design, no formal risk-of-bias assessment or quantitative synthesis was performed. This methodological approach inherently carries limitations, including potential selection bias and reduced reproducibility compared with systematic reviews. However, it enables a broader integrative and mechanistic analysis, which is particularly valuable for complex, multifactorial phenomena such as menstrual cycle-related metabolic variability, where hormonal, behavioral, immune, and microbial factors interact dynamically across temporal scales.

## 3. Menstrual Cycle and Nutrient Metabolism: What Is the Link?

### 3.1. An Overview of the Menstrual Cycle

The menstrual cycle is considered a type of infradian rhythm with an average duration of approximately 29 days, although it typically ranges between 21 and 38 days in healthy reproductive-age women [20,21]. Importantly, substantial interindividual and intraindividual variability exists, and cycle length and hormonal amplitude may fluctuate across the reproductive lifespan.

The first day of heavy menstrual flow is considered day 1 of the menstrual cycle and is included in the follicular phase, which approximately lasts until ovulation (commonly on day 14); the early follicular phase is marked by relatively low concentrations of both E2 and P4, followed by a progressive rise in E2 during the late follicular phase, culminating in ovulation [22]. During this period, FSH promotes the maturation of a cohort of ovarian follicles and stimulates ovarian production of E2 and inhibin B. Rising E2 levels drive endometrial regeneration, vascular remodeling, stromal and glandular proliferation, while inhibin B-mediated negative feedback progressively suppresses FSH, leading to selection of a single dominant follicle. As E2 concentrations increase across the late follicular phase, endocrine feedback transitions from inhibitory to stimulatory, ultimately triggering the mid-cycle LH surge that induces final oocyte maturation and ovulation [22].

Following ovulation, corpus luteum formation establishes a P4-dominant luteal phase, accompanied by a secondary, moderate rise in E2 and suppression of GnRH secretion. This luteal phase corresponds to the endometrial secretory phase, during which P4 promotes glandular differentiation, glycogen accumulation, and increased vascular support within the endometrium, creating a metabolically active tissue prepared for potential implantation [23]. P4 also induces systemic physiological changes, including increased basal body temperature and alterations in cervical mucus properties [24,25]. Importantly, P4-induced slowing of hypothalamic GnRH pulse frequency during the luteal phase alters downstream pituitary signaling dynamics, reinforcing the cyclical alternation between estrogen-dominant and P4-dominant endocrine states [26]. These systemic adaptations reflect the broad distribution of P4 receptors across multiple tissues, including the central nervous system, liver, skeletal muscle, adipose tissue, and gastrointestinal tract.

In circulation, E2 and P4 are transported primarily bound to plasma proteins, including sex hormone-binding globulin (SHBG) and albumin, with only a small fraction existing in the biologically active free form [27]. Fluctuations in SHBG concentrations—modulated by metabolic status, insulin levels, and hepatic function—can therefore influence the proportion of free E2 available for receptor activation, adding an additional regulatory layer to phase-dependent hormonal effects [28,29]. Once at the tissue level, E2 exerts its actions through estrogen receptors α and β (ERα and ERβ), as well as membrane-associated receptors mediating rapid non-genomic signaling [30,31]. P4 signals primarily through progesterone receptor isoforms A and B (PR-A and PR-B), which differentially regulate transcriptional programs [32]. Through these receptor-mediated mechanisms, ovarian steroids influence gene expression, enzyme activity, substrate transporters, mitochondrial dynamics, and inflammatory signaling pathways in metabolically active tissues.

In the absence of fertilization, declining LH support leads to corpus luteum regression and a rapid fall in P4 and E2 levels. This abrupt hormonal withdrawal triggers endometrial shedding (menstruation) and relieves negative feedback at the HPO axis, allowing FSH levels to rise and initiate recruitment of a new follicular cohort, thereby completing the cycle [23]. Menstruation itself represents a transient inflammatory and metabolically demanding state, characterized by localized immune activation and tissue remodeling, with potential systemic metabolic implications [33,34].

Despite this intrinsic temporal organization, current nutritional and metabolic guidelines rarely account for the cyclical nature of female physiology, highlighting a critical gap between endocrine biology and applied metabolic research. This oversight likely contributes to inconsistent findings across studies and limits the translational applicability of metabolic research in women, particularly when menstrual phase is not rigorously verified or considered in study design and data interpretation [35,36]. Given that ovarian hormones dynamically interact with core metabolic pathways as well as with neuroendocrine, immune, microbial, and behavioral systems, a mechanistic, multi-system perspective is required to understand how nutrient metabolism is regulated across the menstrual cycle, as detailed in the following section.

### 3.2. Mechanisms Linking Menstrual Cycle and Nutrient Metabolism

Nutrient metabolism in women is governed by conserved molecular pathways shared across sexes, including insulin signaling, nutrient-sensing kinases, and mitochondrial bioenergetic networks [37]. However, in women, these core pathways are dynamically modulated by ovarian steroids and gonadotropins, resulting in context-dependent metabolic phenotypes that vary across the menstrual cycle and throughout the lifespan [8,38,39]. Thus, the menstrual phase represents a physiological state of shifting endocrine tone that recalibrates substrate utilization without altering the fundamental architecture of metabolic regulation.

Beyond direct endocrine regulation, nutrient metabolism across the menstrual cycle is shaped by the coordinated interaction of neuroendocrine stress responses, immune signaling, gut microbial activity, intestinal barrier function, and behavior, among other factors [40,41]. These systems do not operate independently of ovarian hormones; rather, they are rhythmically tuned by cyclical fluctuations in E2 and P4, amplifying or buffering their metabolic effects and contributing to interindividual variability in metabolic phenotypes [8]. The following sections summarize the principal mechanisms:

(A). Effect of ovarian steroids

Variations in E2 and P4 influence carbohydrate and lipid metabolism through coordinated effects on insulin sensitivity, glucose transport (e.g., GLUT4 translocation), hepatic glucose output, lipolysis, fatty acid oxidation, and mitochondrial efficiency [8,42,43,44,45,46]. Importantly, accumulating evidence suggests that the relative balance between E2 and P4—and not only their absolute circulating concentrations—may act as a key determinant of phase-specific metabolic phenotypes [47,48], particularly in skeletal muscle, liver, and adipose tissue [49,50].

At the cellular level, the integration of nutrient availability and hormonal signaling converges primarily on three molecular networks: (i) insulin and PI3K–Akt signaling, governing glucose uptake and anabolic metabolism; (ii) AMP-activated protein kinase (AMPK), sensing energetic stress and promoting catabolic flux; and (iii) mechanistic target of rapamycin complex 1 (mTORC1), coordinating amino acid availability with protein synthesis [51].

E2 modulates these pathways through both genomic and rapid non-genomic mechanisms. Activation of estrogen receptors (ERα and ERβ) has been shown to enhance insulin signaling efficiency, improve mitochondrial biogenesis via PGC-1α–related pathways, and promote lipid oxidation in metabolically active tissues [42]. In contrast, P4 signaling through PR-A and PR-B isoforms may attenuate insulin sensitivity in certain contexts, alter substrate preference, and influence thermogenic and ventilatory responses [52]. These effects are tissue-specific and influenced by receptor density, co-regulator expression, and intracellular energy status.

Mitochondria function as the central effector organelle, translating these regulatory signals into ATP production, reactive oxygen species generation, redox balance, and metabolic flexibility [6]. Cycle-dependent modulation of mitochondrial dynamics—including biogenesis, fusion–fission balance, and oxidative phosphorylation efficiency—likely contributes to phase-specific differences in substrate utilization observed during rest and exercise, and in health and disease as well [53,54,55].

While these direct endocrine effects form the core biological axis, their metabolic expression is further modulated by higher-order regulatory systems that integrate stress signaling, immune tone, microbial ecology, and behavioral adaptation.

(B). Psychoneuroendocrine regulation beyond ovarian steroids

Psychoneuroendocrine mechanisms constitute a central modulatory layer linking menstrual cycle phase to nutrient metabolism. Cyclical changes in E2 and P4 alter hypothalamic–pituitary–adrenal (HPA) axis sensitivity, modifying cortisol dynamics, stress perception, and emotional reactivity across the cycle [56]. Various systematic reviews and meta-analyses [57,58] reported that cortisol levels appear to be higher in the follicular versus luteal phase, whereas another systematic review and meta-analysis conducted by Klusmann et al. [59] found greater cortisol reactivity to stress in the luteal phase. These findings reflect different dimensions of HPA axis function, with phase-dependent differences in basal activity and stress responsiveness. However, these phase-dependent shifts influence nutrient metabolism indirectly by reshaping insulin sensitivity, gastrointestinal function, and reproductive axis signaling, and by modulating motivational states that govern food intake and dietary choice [60,61,62,63]. In particular, luteal-phase stress sensitivity and neurotransmitter fluctuations interact to promote hedonic food-seeking behavior, thereby altering the quantity and composition of nutrients entering the metabolic system [64].

Circadian rhythms represent an additional regulatory layer interacting with menstrual cycle physiology, with bidirectional influences between ovarian steroids and circadian outputs. In the luteal phase, basal body temperature is elevated and the amplitude of circadian rhythms in temperature, melatonin, and cortisol appears reduced, while subjective sleep quality tends to decline around menses despite relatively stable sleep architecture [65]. Ovarian hormones may also modulate sleep–wake timing and rhythm robustness, with greater circadian stability observed during the follicular phase, potentially linked to estrogen-related effects [66]. Conversely, circadian disruption—such as shift work or irregular sleep patterns—has been associated with menstrual irregularities and altered cycle length, likely through effects on neuroendocrine and metabolic regulation [65]. Together, these findings suggest that circadian–menstrual interactions may contribute to variability in metabolic regulation, although direct evidence linking these rhythms to nutrient metabolism remains limited.

In parallel, the gut microbiota—especially bacterial taxa expressing β-glucuronidase activity within the so-called estrobolome—represents a modulatory interface between endocrine signaling and nutrient metabolism. Through deconjugation reactions, microbial β-glucuronidase can influence the enterohepatic recirculation of estrogens, potentially altering circulating E2 availability [67,68]. However, most supporting evidence currently arises from observational associations and mechanistic models in rodents or small human cohorts, and causal links in large human populations remain under active investigation [69]. Thus, alterations in microbial E2 metabolism may modulate hormone levels rather than act as a primary driver of steroid dynamics. Nevertheless, data in healthy cycling women are limited, and findings should be interpreted cautiously until replicated in larger, well-controlled human studies.

Microbial metabolites such as short-chain fatty acids (SCFAs) and tryptophan-derived ligands further modulate intestinal barrier integrity, immune tone, and nutrient absorption efficiency [70]. Disruptions of this microbial–endocrine axis, whether through dysbiosis, inflammation, or lifestyle factors, can therefore uncouple hormonal signals from their expected metabolic effects [70,71]. However, human evidence linking SCFA and metabolite fluctuations specifically to menstrual phases is still emerging and requires further longitudinal investigation.

Intestinal barrier function represents an additional cycle-sensitive regulatory node. E2 enhances epithelial tight junction integrity via estrogen receptor-mediated mechanisms, promoting selective nutrient absorption and limiting endotoxin translocation [72,73], whereas P4-dominant states may be associated with transient increases in permeability according to some studies [74]. Therefore, while cyclical variation in gut permeability is a mechanistically plausible contributor to systemic immune and metabolic signaling, the evidence in naturally cycling women should be regarded as provisional pending replication in larger cohorts.

In parallel, baseline immune tone varies dynamically across the menstrual cycle, reflecting phase-specific immunological programming rather than a uniform inflammatory or tolerant state [10]. In the menstrual and early follicular phases, endometrial breakdown and tissue shedding are associated with local innate immune activation and a transient pro-inflammatory profile, characterized by elevated neutrophil infiltration and increased pro-inflammatory cytokines like interleukin 6 (IL-6), IL-8 and IL-1β [75,76]. The mid-to-late follicular phase is characterized by a moderately pro-inflammatory milieu that supports follicular maturation and vascularization (e.g., elevated IL-8 and soluble IL-6 receptor), with estradiol promoting a Th2 bias and natural killer (NK) cell cytotoxicity; this transitions periovulatorily into LH-driven recruitment of uterine NK precursors followed by P4 influence, marked by regulatory T cell expansion, increased IL-10 and TGF-β, and 2–3-fold endometrial NK proliferation to support implantation, alongside stabilized or downregulated systemic IL-6/TNF-α [10,77,78,79,80]. Luteolysis reactivates pro-inflammatory signaling with neutrophil and macrophage recruitment, thereby cycling immunological balance between follicular repair/invasion and luteal tolerance [81].

These phase-specific immune shifts impact systemic nutrient metabolism through modulation of insulin signaling, hepatic acute-phase responses, and micronutrient trafficking. For example, increased IL-6 in the early follicular phase can acutely reduce insulin sensitivity and shift iron distribution via hepcidin induction [82], whereas luteal-phase upregulation of anti-inflammatory pathways may support improved lipid handling in some tissues [83]. Taken together, the immune signature of each cycle phase does not simply facilitate reproductive processes but also intersects with systemic nutrient regulation. These immune–metabolic interactions are further modulated by gut-derived signals and intestinal permeability, reinforcing the integrated psychoneuroimmunoendocrine regulation of nutrient handling across the menstrual cycle.

Finally, behavioral and energy-balance mechanisms provide a dynamic interface between internal metabolic regulation and the external nutritional environment. Menstrual-cycle-dependent shifts in appetite, food preference, physical activity patterns, and energy expenditure modulate nutrient availability and metabolic demand, shaping how conserved metabolic pathways are engaged in each phase [84,85,86,87]. When energy availability becomes insufficient relative to expenditure, reproductive and metabolic regulation converge, prioritizing survival over reproductive investment. In this context, low energy availability (LEA) represents a well-characterized physiological state in which insufficient caloric intake relative to exercise and basal demands induces coordinated endocrine and metabolic adaptations [88]. When sustained, LEA may progress to relative energy deficiency in sport (REDs), a broader syndrome involving disruptions in metabolic rate, menstrual function, immune response, bone health, and cardiovascular physiology [89]. At the severe end of this spectrum, persistent energy deficiency may lead to functional hypothalamic amenorrhea (FHA), characterized by suppression of GnRH pulsatility and consequent disruption of ovulatory function, representing a reversible but clinically significant manifestation of energy-conserving neuroendocrine adaptation [90].

Recent systematic evidence indicates that LEA is highly prevalent among athletes (~44% overall), with a substantial proportion also at risk of REDs [88]. Importantly, LEA has been associated with impairments in endurance capacity, training adaptation, neuromuscular performance, and cognitive function, as well as increased susceptibility to illness and bone stress injuries [91]. From a mechanistic perspective, these outcomes are mediated by multi-system endocrine adaptations, including suppression of the HPG axis, alterations in insulin and glucose-regulating hormones, reduced triiodothyronine (T3), elevated cortisol, and disruptions in growth hormone (GH) and insulin-like growth factor 1 (IGF-1) signaling [92].

Within the context of the menstrual cycle, LEA and REDs may override or amplify normal phase-dependent metabolic variability, complicating the interpretation of hormonal and metabolic patterns [93,94]. These conditions highlight the importance of energy availability as a critical upstream regulator of nutrient metabolism, capable of modulating or even uncoupling the expected interactions between ovarian steroids and metabolic pathways. Accordingly, energy availability should be considered a key contextual factor when evaluating menstrual cycle-related metabolic dynamics, particularly in physically active populations.

**Figure 2:**
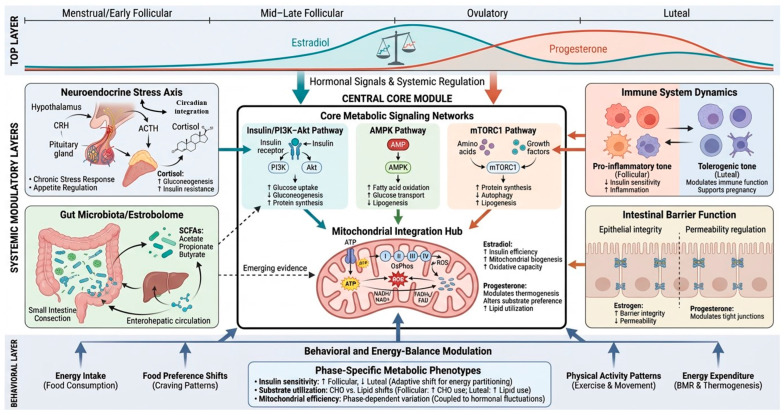
Hormonal and psychoneuroimmunoendocrine regulation of nutrient metabolism throughout the menstrual cycle. Schematic representation of the multi-level regulation of nutrient metabolism across menstrual cycle phases. Fluctuations in ovarian hormones—estradiol (E2) and progesterone (P4)—drive phase-dependent metabolic adaptations. Central metabolic signaling pathways, including insulin/PI3K–Akt, AMP-activated protein kinase (AMPK), and mechanistic target of rapamycin complex 1 (mTORC1), converge at the mitochondrial level, regulating oxidative phosphorylation (OXPHOS), ATP production, and reactive oxygen species (ROS) signaling. Systemic modulators include the hypothalamic–pituitary–adrenal axis (HPA), gut microbiota/estrobolome (short-chain fatty acids, SCFAs), immune dynamics (pro-inflammatory vs. tolerogenic states), and intestinal barrier function. Behavioral factors—energy intake, substrate utilization, physical activity, and energy expenditure (basal metabolic rate, BMR)—interact with these systems to shape metabolic flexibility across phases. Abbreviations: E2, estradiol; P4, progesterone; PI3K, phosphoinositide 3-kinase; Akt, protein kinase B; AMPK, AMP-activated protein kinase; mTORC1, mechanistic target of rapamycin complex 1; OXPHOS, oxidative phosphorylation; ROS, reactive oxygen species; HPA, hypothalamic–pituitary–adrenal axis; SCFAs, short-chain fatty acids; BMR, basal metabolic rate; I-IV: Components of the electron chain transporters.

Figure 2 summarizes the integrated mechanisms linking menstrual cycle dynamics with nutrient metabolism.

## 4. Changes in Macronutrient Metabolism Across Menstrual Cycle

### 4.1. Carbohydrates and Glucose Homeostasis

In eumenorrheic women without overt metabolic disease, the contemporary literature converges on a consistent—though modest—pattern of cyclical variation in insulin sensitivity and glucose handling across the menstrual cycle. From an endocrine perspective, experimental and animal data consistently indicate that E2 exerts insulin-sensitizing effects, whereas P4 tends to antagonize insulin action [4]. In humans, however, this antagonism is only partially expressed and shows substantial heterogeneity, reflecting the integration of ovarian hormone signaling with behavioral, metabolic, and environmental modifiers. Importantly, the absolute magnitude of these differences is small, shows substantial interindividual variability, and is strongly modulated by body mass index (BMI), physical fitness, habitual physical activity, dietary intake, sleep, and likely gut microbial and psychosocial factors [95,96,97]. Thus, in healthy and physically active women, menstrual cycle-related shifts in glucose metabolism typically represent fine-tuning around a metabolic set point largely determined by classical cardiometabolic risk factors.

Classical studies using oral glucose tolerance tests (OGTTs) illustrate both the main tendencies and the methodological limitations of early work. Schieren et al. [95] observed that from nine OGTT studies performed in healthy women between 1975 and 2020, five reported no significant phase-dependent differences, whereas the remainder observed significant changes. Three of them found lower post-load glucose concentrations and area under the curve during the early–mid follicular phase and higher values following ovulation and during the luteal phase, whereas one showed the opposite result. Overall, the authors concluded that if a true physiological effect exists, glucose tolerance appears slightly more favorable in the early cycle and declines after ovulation, although the strength of evidence remains limited. Heterogeneity in glucose loads, imprecise cycle phase classification, and insufficient control of diet and physical activity likely account for the inconsistent findings across this literature.

More recent data from continuous glucose monitoring (CGM) in free-living women support the presence of subtle but reproducible luteal-phase elevations in glycemia. One study conducted in 49 healthy women (with 149 cycles and 554 phases accounted), reported a significant increase in glucose levels during the luteal phase and a minimum in the late follicular phase, although the absolute differences in mean glucose were small despite being statistically robust [98].

In women with type 1 or type 2 diabetes, this same cyclical pattern appears amplified. Multiple cohorts and clinical observations indicate a higher tendency to hyperglycemia and increased insulin requirements during the luteal phase, alongside a greater risk of hypoglycemia in the follicular phase if insulin doses are not adjusted [99]. Similarly, another study with 179 women with T1D obtained similar results, also observing that this increase was independent of exercise duration, total daily insulin units, or reported carbohydrate intake [100], although not all women with T1D exhibit identical patterns, underlining considerable interindividual variability.

Studies using more rigorous techniques to quantify insulin sensitivity have been particularly informative. Using a frequently sampled intravenous glucose tolerance test (IVGTT) with minimal model analysis, Escalante Pulido et al. [97] demonstrated a substantial reduction in the insulin sensitivity index from the follicular to the luteal phase in healthy young women, with unchanged glucose effectiveness and only modest, non-significant increases in acute insulin response. These findings suggest partial—but incomplete—β-cell compensation and support the concept of a relatively more insulin-resistant metabolic milieu during the luteal phase.

In large-scale population studies, similar conclusions are extracted. An analysis of 1906 premenopausal women from NHANES (1999–2006) using cosinor models revealed modest but statistically significant cyclical rhythmicity in fasting glucose, insulin, HOMA-IR, and an index of adipose tissue insulin resistance (ADIPO-IR) after adjustment for BMI, physical activity, or cardiorespiratory fitness [96]. Peaks in HOMA-IR and ADIPO-IR tended to occur during the luteal timeframe. Importantly, the amplitude of these oscillations was greater in women with higher BMI and/or lower fitness, indicating that classical cardiometabolic risk factors amplify hormonally driven fluctuations. However, another study from the same cohort analyzing 1256 participants only observed this rhythmicity for fasting insulin and HOMA-IR, but not for fasting glucose [101]. Age further modified these associations, with younger and older women showing distinct patterns, underscoring the multifactorial nature of cycle-related glucose variability.

Beyond peripheral tissues, central insulin sensitivity also appears to vary across the cycle. A recent study combining intranasal insulin administration with a hyperinsulinemic–euglycemic clamp and functional MRI demonstrated that, in the follicular phase, insulin enhanced both whole-body glucose disposal and hypothalamic responsiveness [102]. In contrast, these effects were absent in the luteal phase, suggesting a state of relative central insulin resistance that may contribute to the observed systemic reduction in insulin sensitivity during this phase.

Cycle-dependent shifts in substrate utilization further shape carbohydrate metabolism at rest and during exercise. While most studies report no major differences in resting energy expenditure or substrate oxidation between phases, differences in substrate storage and utilization during exercise have been described. Several studies suggest greater muscle glycogen synthesis and storage at rest during the luteal phase and a glycogen-sparing effect during submaximal exercise, although findings are not entirely consistent [103]. This phenomenon could be due to the relatively elevated E2 in combination with P4 during the luteal phase, which may augment muscle glycogen storage capacity relative to the low-estrogen environment of the early follicular phase [104]. Alternatively, increased carbohydrate intake during the luteal phase may contribute to higher glycogen availability, suggesting that behavioral factors may partly explain these observations [103].

Tracer studies describe reduced glucose turnover (Ra and Rd) and attenuated hepatic glucose output during luteal-phase submaximal exercise, largely attributed to estrogen-mediated increases in lipid oxidation [105,106]. However, these studies are limited by small sample sizes and specific exercise protocols.

Estrogen can equally promote glucose availability and uptake into type I muscle fibers, providing the fuel of choice during short-duration exercise; an action that can be inhibited by P4 [104]. Notably, these metabolic shifts appear to be intensity-dependent; once exercise exceeds the lactate threshold, the physiological demand for carbohydrate oxidation tends to override the regulatory influence of estrogen [107]. In alignment with these substrate shifts, the peak lactate response to intensive exercise is significantly lower during the luteal phase, a phenomenon attributed to reduced lactate appearance rates and slower carbohydrate turnover compared to the follicular phase [103]. Furthermore, increased leucine oxidation during the luteal phase has been identified as a potential secondary mechanism contributing to this carbohydrate-sparing effect [108].

Despite these mechanistic differences, the practical relevance of endogenous hormonal shifts appears highly context dependent. When carbohydrate availability is sufficient—either through habitual intake or deliberate carbohydrate loading—differences in baseline glycogen stores between phases largely disappear [104]. Moreover, Bailey et al. [109] demonstrated that carbohydrate supplementation during exercise prolonged time to exhaustion similarly in both phases, with no significant phase-specific ergogenic effect. Although some luteal-phase increases in plasma amino acids suggested greater protein catabolism, acute responses to carbohydrate ingestion were largely phase-independent. Collectively, these observations indicate that while ovarian hormones modulate endogenous substrate partitioning, the availability of exogenous carbohydrate in the diet can largely override these subtle phase-dependent differences.

Energy intake and appetite also exhibit cyclical variation with direct implications for carbohydrate exposure. A systematic review of dietary energy intake across the menstrual cycle reports that, in studies with rigorous phase verification (hormone assays or urinary LH), daily energy intake is typically higher in the luteal phase than in the follicular phase, with mean differences ranging from approximately 159 to 529 kcal/day [84]. Some studies found no significant differences, but in others, the excess intake in the luteal phase was particularly evident during weekends [84,110].

Several investigations noted a shift in macronutrient composition, with higher proportions of fat and simple sugars in the luteal phase and a relative reduction in complex carbohydrates, although not always reaching statistical significance and depending on different factors such as physical activity levels [111,112,113]. E2 appears to exert anorexigenic effects, whereas P4 in the presence of E2 tends to increase appetite and preference for energy-dense foods, an effect further modulated by changes in leptin, ghrelin, peptide YY (PYY), and possibly central insulin signaling [114].

Resting energy expenditure generally increases after ovulation, reaching levels up to 5–10% higher in the luteal phase in many women [115,116,117]. Therefore, part of the increased energy intake observed in the luteal phase may represent a compensatory physiological response to elevated metabolic demands rather than purely hedonic overconsumption. Consequently, changes in diet quantity and quality may contribute as much as, or more than, direct hormonal effects to observed phase-related glycemic variation.

The gut microbiota introduces an additional—though incompletely defined—layer of regulation. Current human data in healthy women are scarce and largely correlative, and no robust menstrual phase-specific microbiota signature has been established. Mechanistically, however, several microbiota–hormone–glucose axes are plausible. The estrobolome may modulate circulating estrogen levels and thereby indirectly influence glucose metabolism across the cycle. Additionally, short-chain fatty acids (SCFAs) such as butyrate, propionate, and acetate influence intestinal permeability, low-grade inflammation, incretin secretion, and satiety signaling [118,119].

P4 and E2 also affect gastrointestinal motility and hormone secretion. Previous studies indicate that colonic transit time and gastric emptying can vary across the cycle, likely through P4-mediated changes in smooth muscle function and crosstalk with gastrointestinal hormones [120]. Low doses of P4, similar to luteal-phase levels, have been associated with accelerated gastric emptying and higher postprandial peaks of glucose, insulin and GLP-1, whereas higher levels of P4 slow transit and may worsen glucose tolerance in susceptible individuals [120]. Given that transit time shapes microbial composition, a bidirectional and potentially cycle-dependent interaction between hormones, motility, microbiota, and incretin physiology is plausible, although human evidence remains preliminary.

The role of the microbiota is better documented in pathological contexts such as polycystic ovary syndrome (PCOS) and menstrual disorders. In women with PCOS and/or obesity, characteristic dysbiotic patterns—often including increased Firmicutes and decreased Bifidobacterium, Akkermansia and Faecalibacterium—are associated with systemic insulin resistance and ovarian dysfunction [121]. In more detail, the association between dysbiosis and insulin resistance is thought to be connected with the enhanced intestinal permeability and endotoxemia, associated with chronic low-grade inflammation, along with alterations in microbiota-derived metabolites—including short-chain fatty acids (SCFAs), bile acids, and branched-chain amino acids (BCAAs)—which collectively may influence glucose homeostasis and energy balance [121]. However, most evidence remains observational or translational, and causal relationships require further validation. Interventional trials using prebiotics and probiotics in PCOS have demonstrated improvements in insulin sensitivity and sometimes in menstrual regularity, suggesting that modifying the microbiota can beneficially influence both metabolism and HPO axis function [122]. However, these findings cannot yet be extrapolated to conclude that physiological cycle-related microbiota fluctuations meaningfully drive carbohydrate metabolism in healthy women; rather, they highlight the gut ecosystem as a potential modulator under conditions of metabolic perturbation. Consistent with this context-dependent framework, lifestyle factors further contribute to metabolic heterogeneity in PCOS, as dietary patterns characterized by higher fat intake, greater consumption of medium/high glycemic index foods, and lower physical activity have been associated with increased insulin resistance and inflammation, whereas higher fiber intake shows protective associations with metabolic markers [123]. These findings support the notion that dietary composition and energy balance interact with endocrine and microbiota-related mechanisms, reinforcing metabolic heterogeneity in PCOS and highlighting the importance of lifestyle context in modulating glucose homeostasis.

**Figure 3:**
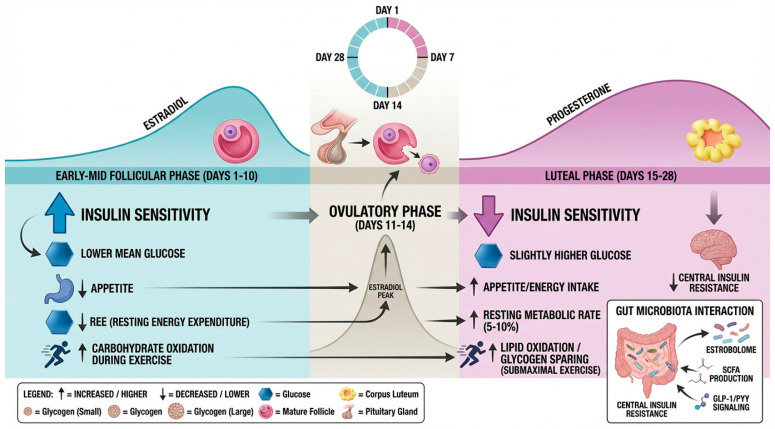
Schematic representation of metabolic changes across menstrual cycle phases. During the early–mid follicular phase, characterized by rising estradiol (E2), there is increased insulin sensitivity, lower mean glucose levels, reduced appetite, and a predominance of carbohydrate oxidation during exercise. The ovulatory phase represents a transitional metabolic state associated with peak E2 levels. In the luteal phase, dominated by progesterone (P4), insulin sensitivity decreases, glucose levels are slightly elevated, appetite and energy intake increase, and resting energy expenditure (REE) rises. This phase is also associated with increased lipid oxidation and glycogen sparing during submaximal exercise, as well as interactions with gut microbiota and estrobolome activity. Abbreviations: E2, estradiol; P4, progesterone; REE, resting energy expenditure; SCFAs, short-chain fatty acids; GLP-1, glucagon-like peptide-1; PYY, peptide YY.

In Figure 3, we summarize the main findings and mechanisms connecting the menstrual cycle with carbohydrate metabolism.

### 4.2. Lipid Metabolism

Lipid metabolism exhibits modest but consistent cyclical variations across the menstrual cycle in eumenorrheic women, primarily driven by fluctuations in E2 and P4. Plasma lipoprotein profiles show phase-dependent patterns. Total cholesterol (TC) and low-density lipoprotein cholesterol (LDL-C) tend to peak during the follicular phase and decline during the luteal phase, coinciding with elevated E2 and P4 levels [124]. High-density lipoprotein cholesterol (HDL-C) typically reaches its highest concentrations during the late follicular or periovulatory phase, near the E2 surge, though this peak may be missed in studies comparing only broad follicular versus luteal averages [124,125]. Triglycerides (TG) often display higher mean levels during the follicular phase (by approximately 7.4% in controlled studies), with lower HDL-C (by about 5.8%), though these shifts are less consistently significant than those for TC and LDL-C [83].

Population-level data reinforce these trends. NHANES analyses indicate cyclical rhythmicity in triglycerides and HDL-C after adjusting for confounders, with greater amplitudes in women with higher BMI or lower fitness [96]. However, these oscillations remain quantitatively small and highly heterogeneous, partly due to small sample sizes, variable phase verification (self-report vs. hormonal confirmation), and confounding dietary or behavioral factors. In pathological states such as PCOS, dyslipidemia amplifies these patterns, and luteal-phase improvements appear blunted [126].

Indices of insulin resistance (e.g., HOMA-IR, ADIPO-IR), low-grade systemic inflammation, and adipokine profiles co-vary with menstrual phase and partly mediate lipid fluctuations [96]. Thus, menstrual-cycle-related changes in lipids likely reflect the integrated action of ovarian steroids, insulin sensitivity, adipose tissue function, and lifestyle factors rather than isolated hormonal effects.

Recent evidence further explores the potential roles of specific dietary fatty acids in these cyclical dynamics. Omega-3 polyunsaturated fatty acids (PUFAs; EPA and DHA) supplementation (2–3 g/day) has been associated with reductions in FSH concentrations (≈19–30%) in normal-weight eumenorrheic women, particularly during the follicular phase, without consistent changes in LH or ovarian steroids [127]. These findings suggest a possible modulatory effect on HPO signaling, potentially mediated by anti-inflammatory pathways, although causality and long-term reproductive implications remain uncertain. Notably, such effects appear attenuated in obesity and metabolic syndrome.

Conversely, high omega-6:omega-3 ratios (>10:1) are associated with ovulatory infertility and longer cycles (OR 1.8), driven by pro-inflammatory eicosanoids (e.g., PGE2 from arachidonic acid), exacerbated luteally by P4 [128]. However, these data are largely observational and do not establish a direct mechanistic link between fatty acid ratios and ovulatory dysfunction. Monounsaturated fatty acids (MUFAs, e.g., oleic acid) from Mediterranean diets support favorable TC/HDL-C ratios and membrane fluidity, correlating with shorter cycles and lower dysmenorrhea, while trans-fatty acids (TFAs) replacing MUFAs/PUFAs elevate infertility risk independently of BMI/hormones [128,129]. Whether these associations directly modify luteal lipid oxidation or HPO feedback remains plausible but incompletely demonstrated in healthy populations [130].

Ratios indicative of cardiovascular risk, such as TC/HDL-C, LDL-C/HDL-C, and TG/HDL-C, are generally lower (more favorable) during the luteal phase compared to the follicular phase ([131]). E2 appears to mediate many of these changes through rapid effects on HDL-C elevation (e.g., via reduced hepatic lipase activity) and longer-term reductions in TC and LDL-C (e.g., via upregulated LDL receptors), whereas P4 may exert counter-regulatory or anti-estrogenic actions [132]. For instance, in the BioCycle Study (*n* = 259 cycles in 62 women), E2 was positively associated with HDL-C in acute effects models and inversely associated with TC, LDL-C and TG in persistent effects models [125]. A cross-sectional analysis of biomarkers confirmed higher pro-atherogenic non-HDL-C, ApoB, and ApoB/HDL ratios during the low-estrogen follicular phase, with remnant cholesterol (a TG-rich lipoprotein marker) elevated luteally and uncorrelated with estrogen [126].

However, absolute differences are small (e.g., 5–10% for TC/LDL-C), with intraindividual variability up to 8–19% for TC, and clinical misclassification risks are low unless cycle phase is ignored in repeated lipid panels [124]. Although more women exceed clinical cholesterol thresholds during the follicular phase than during the luteal phase (14.3% vs. 7.9%), only a minority remain persistently elevated [133]. Therefore, while cycle phase may influence single lipid measurements, the risk of long-term clinical misclassification appears limited, particularly when repeated measurements are obtained.

Additional changes in lipid metabolism have also been reported in past works. Draper et al. [3] demonstrated various metabolic variations during the menstrual cycle in 34 eumenorrheic, healthy women. Specifically, they observed that a substantial proportion of plasma phospholipids—especially lysophosphatidylcholines (LPCs), phosphatidylcholines (PCs), and lysophosphatidylethanolamines (LPEs)—were significantly decreased in the luteal phase, indicating coordinated modulation of membrane lipid metabolism. Conversely, acylcarnitines in plasma and urine showed a trend toward increased concentrations in the periovulatory phase, suggesting enhanced fatty acid transport and β-oxidation around ovulation. In parallel, emerging data suggest that gut microbiota composition and its metabolites (notably short-chain fatty acids) display modest menstrual-phase-related variation and are associated with markers of dyslipidemia and dysbiosis, implying that microbiota–host interactions may further modulate lipid handling across the cycle, particularly in women with obesity or metabolic syndrome [121,126].

During exercise, luteal-phase fat oxidation rates are often higher than in the follicular phase, supporting a relative glycogen-sparing effect, as previously stated [8]. Despite this, not all studies report a significant increase in fat oxidation during exercise in the luteal phase, particularly at higher intensities (≥75% of VO2max) or in trained athletes [107,134]. Oral contraceptive use may further modify substrate oxidation patterns [135].

Behavioral factors—including changes in physical activity, sleep duration, and dietary intake—also co-vary with cycle phase and lipid concentrations, further complicating attribution to ovarian hormones alone [96,136,137].

**Figure 4:**
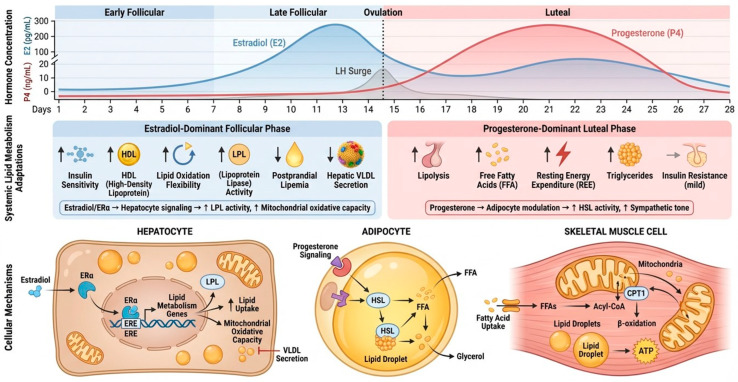
Changes in lipid metabolism throughout the menstrual cycle. Schematic representation of phase-dependent regulation of lipid metabolism. During the estradiol (E2)-dominant follicular phase, increased insulin sensitivity, enhanced lipid oxidation flexibility, and improved lipoprotein metabolism (increased high-density lipoprotein [HDL], reduced low-density lipoprotein [LDL], and lower postprandial lipemia) are observed, supported by estrogen receptor (ER)-mediated signaling and increased mitochondrial oxidative capacity. In contrast, the progesterone (P4)-dominant luteal phase is characterized by increased lipolysis, elevated free fatty acids (FFAs), higher resting energy expenditure (REE), increased triglycerides, and mild insulin resistance, driven in part by hormone-sensitive lipase (HSL) activation and sympathetic tone. At the cellular level, hepatocytes regulate lipoprotein metabolism and very-low-density lipoprotein (VLDL) secretion; adipocytes increase lipolytic activity via HSL-mediated triglyceride breakdown; and skeletal muscle enhances fatty acid uptake and β-oxidation through carnitine palmitoyltransferase 1 (CPT1)-dependent mitochondrial pathways. Abbreviations: E2, estradiol; P4, progesterone; HDL, high-density lipoprotein; LDL, low-density lipoprotein; VLDL, very-low-density lipoprotein; FFAs, free fatty acids; REE, resting energy expenditure; ER, estrogen receptor; HSL, hormone-sensitive lipase; CPT1, carnitine palmitoyltransferase 1.

Taken together, menstrual-cycle-related variations in lipid metabolism appear physiologically consistent but modest in magnitude, and are substantially shaped by metabolic status, lifestyle, and methodological considerations. In Figure 4, a summary of the possible mechanisms and changes related to lipid metabolism throughout the menstrual cycle is represented.

### 4.3. Protein and Amino Acid Metabolism

Protein and amino acid metabolism display subtle cyclical variations across the menstrual cycle in eumenorrheic women, with evidence of a modestly more catabolic state during the luteal phase, potentially driven by P4 and modulated by E2. Plasma amino acid profiles exhibit phase-dependent changes, with concentrations of branched-chain amino acids (BCAAs; leucine, isoleucine, valine), alanine, glutamine, threonine, tyrosine, arginine, ornithine, glycine, methionine, and proline typically lower in the luteal phase compared to follicular and menstrual phases [3,138].

In a metabolomics study of 34 healthy women, 39 amino acids and derivatives decreased significantly in the luteal phase, suggestive of increased anabolism or utilization for P4-driven processes such as endometrial thickening and nitrogen retention [3]. Urea cycle intermediates also decline luteally, consistent with altered nitrogen handling. These patterns are corroborated by targeted profiling showing negative correlations between alanine, glutamine, threonine, and tyrosine levels and P4 fluctuations [138]. Population-level analyses also suggest modest rhythmicity in circulating amino acids [96].

Despite these circulating changes, controlled tracer studies indicate that whole-body and myofibrillar protein synthesis rates remain largely unchanged across menstrual phases [139,140]. This suggests that lower plasma amino acid concentrations during the luteal phase may primarily reflect increased flux, utilization, or redistribution rather than reduced anabolic capacity. Supporting this interpretation, previous studies have reported modestly higher leucine flux and oxidation during the luteal phase [108]. Similarly, lysine requirements assessed through indicator amino acid oxidation methods appear slightly elevated during the luteal phase, consistent with greater amino acid catabolism [141].

During exercise, amino acid oxidation may also increase modestly in the luteal phase, contributing to the small but measurable contribution of amino acids to total energy expenditure. However, carbohydrate remains the dominant substrate at higher exercise intensities, and post-exercise muscle protein synthesis does not appear to differ between menstrual phases [104,140].

Hormonal interactions likely contribute to these patterns. E2 may promote anabolic sparing (e.g., via mTOR signaling in muscle), while P4 drives catabolism for reproductive tissue such as endometrial remodeling, a phenomenon associated with a slight decrease in muscle strength and increased muscle fatigue [142]. Additional metabolic and behavioral factors may also contribute to these dynamics. Phase-related changes in insulin sensitivity, inflammatory mediators, adipokines, and energy intake can influence amino acid turnover and nitrogen balance [96]. However, current evidence does not support formal clinical recommendations for cycle-phase-specific protein intake in healthy women. While slightly higher protein intake during the luteal phase has been proposed in certain contexts (e.g., athletic populations), existing evidence remains limited and requires further controlled investigation [46].

**Figure 5:**
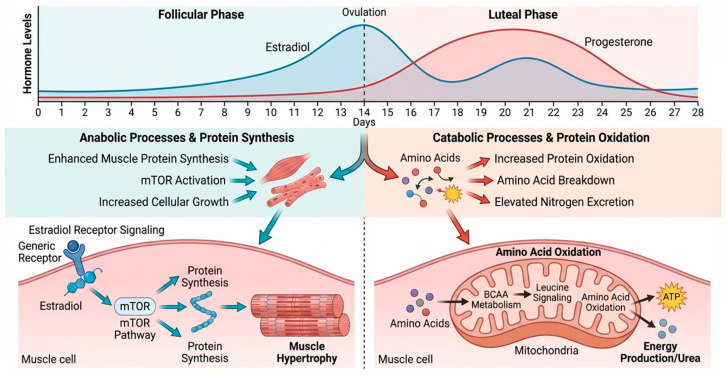
Changes in amino acid and protein metabolism throughout the menstrual cycle. Schematic representation of phase-dependent regulation of protein metabolism. During the estradiol (E2)-dominant follicular phase, anabolic processes are enhanced, including increased muscle protein synthesis, mechanistic target of rapamycin (mTOR) activation, and cellular growth, mediated by estrogen receptor (ER) signaling. In contrast, the progesterone (P4)-dominant luteal phase is characterized by a shift toward catabolic processes, including increased protein oxidation, amino acid breakdown, and elevated nitrogen excretion. At the cellular level, E2 promotes mTOR pathway activation and muscle protein accretion, whereas in the luteal phase, increased amino acid oxidation occurs at the mitochondrial level, involving branched-chain amino acid (BCAAs) metabolism, leucine signaling, and ATP production. Abbreviations: E2, estradiol; P4, progesterone; mTOR, mechanistic target of rapamycin; ER, estrogen receptor; BCAA, branched-chain amino acids; ATP, adenosine triphosphate.

Overall, available data indicate that protein metabolism across the menstrual cycle undergoes subtle physiological modulation (Figure 5), with a tendency toward increased amino acid flux and oxidation during the luteal phase, while net anabolic capacity appears largely preserved. In Supplementary Table S1, a summary of the main findings observed in macronutrient metabolism according to the menstrual cycle is presented.

## 5. Changes in Micronutrient Metabolism Across Menstrual Cycle

### 5.1. Vitamins

Vitamin metabolism shows measurable—albeit generally modest—interactions with menstrual cycle hormones in eumenorrheic women, with the most consistent human evidence clustering around one-carbon metabolism (folate/B-vitamins and homocysteine) and vitamin D; by contrast, data for vitamins C, A and E are sparse and typically do not support robust phase-dependent variation. Importantly, most reported changes reflect fluctuations in circulating biomarkers rather than demonstrated alterations in whole-body vitamin pools or established dietary requirements.

Within one-carbon metabolism, serum folate is typically lowest during menses and rises into the luteal phase, while plasma homocysteine is lowest in the luteal phase and higher in the follicular/peri-ovulatory window, consistent with hormonally patterned shifts in methyl-group demand, oxidative stress, and inflammatory signaling across the cycle [143]. In the BioCycle Study (259 women followed for up to two cycles with repeated timed sampling), higher serum folate was specifically associated with higher luteal P4 (i.e., corpus luteum function), whereas higher homocysteine was associated with lower E2 across the cycle, higher FSH around expected ovulation, and lower luteal P4 [143]. Importantly, homocysteine at expected ovulation predicted a 33% greater risk of sporadic anovulation, while a higher folate:homocysteine ratio at ovulation was protective, suggesting that maintaining adequate availability of one-carbon cofactors may be most consequential in the peri-ovulatory to early luteal transition when ovulatory competence and luteinization are established [46]. However, these associations should not be interpreted as evidence that within-cycle changes in biomarker concentrations necessarily reflect altered dietary requirements, as plasma volume shifts, inflammatory tone, and insulin sensitivity also vary across the cycle and may influence circulating concentrations independently of intake.

Dietary data from the same cohort reinforce the idea that these B-vitamins function more as metabolic capacity factors than as direct cycle regulators in well-nourished eumenorrheic women. In BioCycle, higher vitamin B2 intake was associated with a small inverse association with E2 and with lower homocysteine, and higher vitamin B12 intake was associated with lower homocysteine (with only small changes in androgens), while B6 intake showed at most a suggestive (non-significant) tendency toward higher FSH; none of B2/B6/B12 intakes predicted sporadic anovulation risk [144]. Collectively, these findings suggest that adequate B-vitamin status supports metabolic resilience across phases, but do not support large phase-specific dosing adjustments in healthy women with sufficient habitual intake.

Vitamin D provides a complementary example, where circulating 25(OH)D often shows minimal follicular–luteal change, yet functional vitamin D biology may still be cycle-responsive. A systematic review of longitudinal studies concluded that most studies report no meaningful within-cycle change in 25(OH)D, while findings for 1,25(OH)_2_D are inconsistent, with some studies showing increases within the follicular phase and/or from the follicular to luteal phases [145]. In a prospective study of vitamin D metabolites and reproductive hormones across 163 cycles, mean 25(OH)D did not differ between luteal and follicular phases, but 1,25(OH)_2_D and iPTH showed small fluctuations and lower vitamin D status was associated with lower mean E2 across the cycle [146]. Given the tight endocrine regulation of 1,25(OH)_2_D by calcium–PTH homeostasis, any apparent menstrual-cycle variation in active vitamin D metabolites is likely secondary rather than a primary driver of reproductive endocrine dynamics. From a needs-oriented standpoint, these data suggest that stable 25(OH)D does not preclude phase-specific downstream signaling interactions; however, current evidence does not justify assuming altered intra-cycle vitamin D requirements in replete women [145,146].

Consistent with this, observational work linking vitamin D status to menstrual characteristics indicates that low 25(OH)D is associated with less favorable cycle dynamics. In a prospective analysis using prospectively recorded menses/ovulation data, lower 25(OH)D was associated with long menstrual cycles and long follicular phases, with a tendency toward short luteal phases, and the strongest associations were observed among women with vitamin D deficiency (<20 ng/mL), but elevations in risk were also noted for 20–<30 ng/mL compared with ≥40 ng/mL [147]. These findings align with a pragmatic message: maintaining adequate vitamin D status may be particularly relevant for women at risk of subtle ovulatory inefficiency or shortened luteal phases, even when overt deficiency is not the focus [147].

Beyond eumenorrheic populations, emerging evidence from clinical conditions further highlights the context-dependent role of water-soluble vitamins in metabolic and endocrine regulation. In women with PCOS, alterations in B-vitamin status have been reported, with lower concentrations of thiamine (B1), riboflavin (B2), pyridoxine (B6), and folate compared to healthy controls, alongside higher circulating vitamin C levels [148]. Importantly, several of these vitamins show significant associations with key metabolic and hormonal parameters, including positive correlations between vitamin C and insulin/testosterone, riboflavin and androgen levels, and pyridoxine with androstenedione, as well as inverse relationships between niacin and both SHBG and HDL. These findings suggest that water-soluble vitamins may be involved in the modulation of oxidative stress, low-grade inflammation, and endocrine dysfunction characteristic of PCOS. However, given the observational nature of these data and the relatively small sample sizes, these associations should be interpreted cautiously and do not establish causality. Rather than indicating phase-specific requirements, this evidence supports the concept that micronutrient status may become more functionally relevant in metabolically dysregulated states, where deficiencies or imbalances could exacerbate underlying pathophysiological processes.

Exercise and energy availability can further amplify apparent “phase needs,” because luteal-phase increases in resting energy expenditure and fat oxidation increase demand on mitochondrial cofactor systems, including B-vitamins (one-carbon and redox) and vitamin D–linked muscle and recovery pathways; however, most sports nutrition guidance treats these as chronic adequacy targets rather than sharp within-cycle dosing changes [149]. Current female-athlete nutrition guidance highlights vitamin D (and folate/iron/calcium) as micronutrients warranting attention, and notes that daily vitamin D supplementation of 1000–2000 IU can be reasonable in athletes depending on baseline status and context [149]. Integrating this with the menstrual-cycle literature, a practical article-level framing is that luteal-phase metabolism (higher thermogenesis and lipid oxidation) plausibly increases functional requirements for B-vitamin cofactors and vitamin D–supported recovery, and that this matters most when baseline intake/status is marginal, when training loads are high, or when metabolic comorbidities (e.g., insulin resistance, obesity, PCOS) are present [144,146].

Finally, for vitamins C, A and E, available longitudinal studies in naturally cycling women report only modest or inconsistent phase-related changes, with variations that are generally small compared with those observed for one-carbon metabolism markers and vitamin D and often fall within normal biological variability [150,151].

### 5.2. Minerals

Mineral homeostasis exhibits pronounced cyclical variations across the menstrual cycle in eumenorrheic women, driven primarily by menstrual blood loss for iron and hormonal regulation of absorption, renal excretion, and tissue demands for trace elements like zinc, magnesium, selenium, and copper. Serum iron biomarkers follow a pattern tightly linked to blood loss and hepcidin–erythropoietin dynamics: hemoglobin (Hb), ferritin (SF), and transferrin saturation (TS) nadir during menses and peak luteally, with soluble transferrin receptor (sTfR) elevated transiently during menses due to increased erythropoiesis [152,153,154]. Hepcidin mirrors iron availability, decreasing during menses and peaking in the early luteal phase [152]. These cyclical changes are physiologically coherent and among the most robust micronutrient fluctuations documented across the menstrual cycle.

Mean ferritin decline per menses has been estimated at ≈10–27 ng/mL, with heavy bleeding substantially increasing anemia risk [154]. Further analyses confirm this rhythmicity, amplified by specific factors such as low dietary iron intake or high BMI. For instance, obese women (BMI > 30) exhibit 15–25% greater menses ferritin drops and higher luteal inflammation-driven hepcidin [155]. Importantly, timing of blood sampling materially influences iron status classification, and sampling during menses may overestimate iron deficiency prevalence due to transient reductions in circulating indices.

Beyond biomarker fluctuations, iron status also has clear functional implications, particularly in physically active women. Recent systematic evidence [156] in high-level female athletes indicates that iron deficiency (serum ferritin < 40 µg/L) is associated with modest but meaningful impairments in endurance performance (~3–4%), as well as potential reductions in maximal aerobic capacity and training adaptation. In addition, iron supplementation (~100 mg/day of elemental iron) has been shown to improve endurance performance (2–20%) and increase maximal aerobic capacity (6–15%) in iron-deficient athletes, even in the absence of overt anemia. However, effects on anaerobic performance and strength outcomes appear more variable, and much of the available evidence is limited by small sample sizes and moderate methodological quality [156]. Accordingly, these findings support the importance of maintaining adequate iron status—particularly in populations exposed to high training loads and menstrual blood loss—while emphasizing the need for individualized assessment rather than routine supplementation.

Trace minerals (zinc, magnesium, selenium, copper) often display phase-related variability, but the magnitude of these shifts is typically modest and their functional significance in well-nourished women remains uncertain. Reported patterns include higher serum zinc around mid-follicular to ovulatory windows with nadirs during menses, and phase-dependent fluctuations in magnesium and selenium with partial rebound during the luteal phase, although findings are not fully consistent across cohorts [155]. However, much of this literature is limited by small sample sizes, observational designs, heterogeneous phase definitions, and incomplete control of diet, physical activity, and ovulation verification, which constrains causal inference.

In a cohort of 45 women, zinc declined by 6.6% (*p* = 0.009) and magnesium by 4.6% (*p* < 0.001) from the early follicular to mid-luteal phase, and the proportion meeting criteria for magnesium deficiency was higher in the luteal phase (29% vs. follicular) [157]. These data support the presence of measurable within-cycle variability, yet do not necessarily indicate clinically meaningful depletion. Mechanistically, selenium is relevant to follicular maturation and redox control (e.g., glutathione peroxidase activity), and copper appears relatively stable in circulation, although lower follicular fluid copper has been linked to poorer in vitro fertilization (IVF) outcomes, suggesting that local (tissue) micronutrient status may be more informative than serum measures alone [158]. Calcium is also relevant in reproductive tissues; controlled trials indicate that calcium supplementation can reduce premenstrual symptoms, supporting a role for adequacy rather than phase-specific cycling per se [159].

Several mechanistic hypotheses link ovarian steroids to trace mineral handling—including estrogen-related regulation of zinc transport and P4-related effects on renal retention—but most supporting evidence is experimental and should be extrapolated cautiously to healthy cycling women [155]. At the population level, lower dietary zinc and magnesium have been associated with menstrual irregularity and sporadic anovulation, but these associations are observational and may be confounded by overall diet quality and metabolic health [160].

During exercise, mineral demands parallel macronutrient and energy shifts. Given luteal-phase increases in resting energy expenditure and lipid oxidation, reliance on magnesium- and zinc-dependent enzymatic systems may plausibly increase, particularly under high training loads or marginal intake. However, direct evidence supporting phase-targeted trace mineral supplementation to improve performance or symptoms remains limited. Existing data instead suggest that training status, overall intake, and recovery behaviors largely determine whether measurable phase-related dips translate into functional effects [161].

Emerging evidence from clinical populations further supports the context-dependent relevance of mineral status. In women with PCOS, alterations in trace elements have been described, with some studies reporting imbalances in minerals such as zinc, magnesium, sodium, and nickel, alongside potential associations with lipid profile disturbances [162]. While absolute differences between PCOS and control groups are often modest, these findings suggest that subtle disruptions in mineral homeostasis may interact with metabolic dysfunction, including insulin resistance and dyslipidemia [162]. However, given the limited sample sizes and observational nature of these studies, such associations should be interpreted cautiously and do not establish causal relationships. Rather, they reinforce the concept that micronutrient status may have greater functional relevance in metabolically altered states, where even small imbalances could contribute to underlying pathophysiological processes.

**Figure 6:**
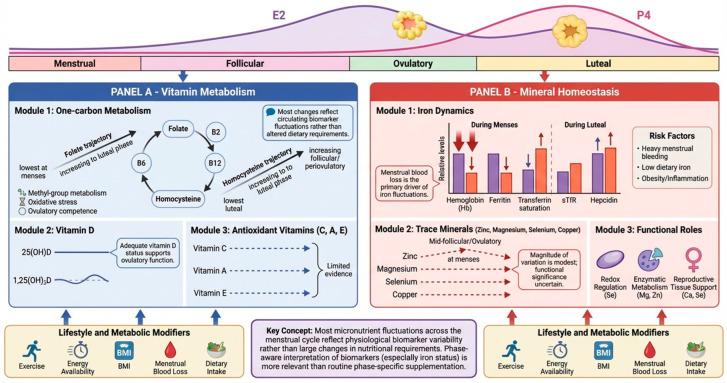
Changes in micronutrient metabolism throughout the menstrual cycle. Schematic representation of phase-dependent changes in vitamin metabolism and mineral homeostasis. Panel A illustrates vitamin-related processes, including one-carbon metabolism (folate, vitamins B6 and B12), vitamin D metabolism, and antioxidant vitamins (C, A, E), which exhibit modest fluctuations primarily driven by hormonal variation and physiological context. Panel B depicts mineral dynamics, highlighting iron metabolism (hemoglobin, ferritin, transferrin saturation, soluble transferrin receptor [sTfR], and hepcidin), with menstrual blood loss as a key determinant, as well as trace elements (zinc, magnesium, selenium, copper), whose variations are generally small and context-dependent. Lifestyle and metabolic modifiers—including exercise, energy availability, body mass index (BMI), menstrual blood loss, and dietary intake—interact with hormonal fluctuations (E2 and P4) to influence micronutrient status and functional roles such as redox balance, enzymatic activity, and tissue support. Abbreviations: E2, estradiol; P4, progesterone; B6, vitamin B6; B12, vitamin B12; sTfR, soluble transferrin receptor; BMI, body mass index.

Taken together, mineral–cycle interactions form an integrative network where menstrual blood loss (iron) and trace element dynamics interact with metabolic status and lifestyle. For clinical and applied practice, the most evidence-based approach is phase-aware interpretation of biomarkers (especially iron indices) and targeted screening of high-risk subgroups (e.g., heavy menstrual bleeding, athletes, obesity/PCOS), rather than routine phase-specific dosing protocols. In Figure 6, changes in micronutrient metabolism across the menstrual cycle are summarized.

## 6. Changes in Bioactive Compounds Across Menstrual Cycle

Bioactive compounds such as phytoestrogens and polyphenols interact with menstrual cycle physiology primarily as modulators of estrogen signaling, oxidative stress, and inflammation, rather than through clearly demonstrated phase-specific endogenous fluctuations or quantifiable requirements. Observational and longitudinal data in eumenorrheic women suggest that habitual intake of isoflavones and lignans exerts modest, context-dependent effects on reproductive hormones and cycle regularity. In a large cohort of healthy women attempting conception (*n* = 1209 cycles), higher urinary isoflavones were not associated with meaningful changes in cycle length but were linked to lower odds of menstrual irregularity (OR 0.79, 95% CI 0.64–0.98), whereas higher lignan excretion showed slightly increased irregularity risk (OR 1.32, 95% CI 1.06–1.64) [163]. In the BioCycle Study (*n* = 259 premenopausal women), greater isoflavone intake was associated with higher follicular SHBG (+8.6%, *p* = 0.05) and lower luteal testosterone (−14.5%, *p* = 0.04), with a trend toward reduced sporadic anovulation (HR 0.56, *p* = 0.07), without impacting E2, P4, FSH, or LH, indicating mild anti-androgenic and SHBG-modulating effects without overt HPO disruption [164]. Mechanistically, these effects stem from phytoestrogens’ weak selective estrogen receptor modulator (SERM) activity, preferentially binding ERβ and influencing estrogen-responsive genes, particularly in estrogen-dominant phases (follicular/ovulatory); however, interindividual variability in metabolism—driven by gut microbiota (estrobolome)—substantially attenuates population-level consistency [165,166].

Polyphenols (flavonoids, phenolic acids) and carotenoids contribute to redox/inflammatory control, with plausible higher functional relevance luteally (increased ROS), but total flavonoid intake shows limited associations with E2, P4, or prolactin in premenopausal women, though low carotenoid status links to irregularity (OR 1.2–1.5) [167]. Gut microbes convert precursors to bioactive metabolites (e.g., equol from daidzein, urolithins from ellagitannins) via β-glucuronidases/β-glucosidases, with P4-enhanced bile flow/SCFAs amplifying recirculation; dysbiosis reduces equol producers (<30%), blunting benefits [168,169]. Lifestyle factors (exercise ROS, luteal fiber appetite) modulate these, supporting diet quality/microbiota health over phase-dosing [170]. In Supplementary Table S2, we summarize changes in micronutrient and bioactive compounds according to the menstrual cycle.

## 7. Limitations of the Review and Research Gaps

Despite providing an integrative and mechanistic overview of nutrient metabolism across the menstrual cycle, several limitations should be acknowledged.

First, a substantial proportion of the mechanistic insights discussed in this review are derived from preclinical models or indirect human evidence. While these data provide valuable biological plausibility, many proposed pathways—particularly those involving mitochondrial function, substrate partitioning, and micronutrient dynamics—remain insufficiently validated in well-controlled human studies. Accordingly, some interpretations should be considered as hypothesized or proposed mechanisms that require further empirical confirmation.

Second, the available human literature is characterized by considerable methodological heterogeneity. Many studies rely on small sample sizes, limiting statistical power and increasing the likelihood of type II errors. In addition, inconsistencies in menstrual cycle phase determination represent a major source of variability. While some studies employ rigorous methods such as hormonal confirmation or ovulation testing, others rely on calendar-based estimations or self-reported cycle phases, which may lead to misclassification and obscure true physiological effects.

Third, variability in study design further complicates interpretation. Differences in exercise protocols, nutritional interventions, metabolic assessment techniques, and participant characteristics (e.g., training status, energy availability, use of hormonal contraceptives) contribute to heterogeneity across findings. These factors likely underlie some of the conflicting results reported in the literature and limit direct comparability between studies.

Fourth, the complex and multi-system nature of menstrual cycle-related metabolic regulation presents an inherent challenge. Interactions between endocrine, immune, microbial, and behavioral systems are dynamic and context-dependent, yet most studies examine these components in isolation. As a result, the integrative framework proposed in this review, while conceptually useful, may not fully capture the temporal and individual variability observed in real-world settings.

Finally, as a narrative review, this work is subject to inherent limitations, including potential selection bias and reduced reproducibility compared with systematic approaches. Although efforts were made to enhance transparency in the literature selection and to prioritize high-quality evidence, the absence of formal risk-of-bias assessment and quantitative synthesis should be considered when interpreting the conclusions.

## 8. An Integrative Perspective and Future Directions

A growing body of work indicates that menstrual cycle-related variability in nutrient metabolism is small-to-moderate in magnitude but may be sufficiently consistent to carry practical relevance for clinical nutrition, exercise prescription, and metabolic risk stratification. Cycle phase appears to influence energy intake, resting energy expenditure, substrate selection, and micronutrient handling, with a tendency toward higher energy intake and greater reliance on lipid oxidation during the mid-luteal phase compared with the early follicular and peri-ovulatory periods [84,171]. Metabolomics data further show coordinated rhythmic shifts in amino acids, B-vitamins, and lipid species across phases, which may reflect windows of greater “metabolic vulnerability” in which nutritional inadequacy, low energy availability, or high training loads may more readily precipitate symptoms or performance decrements [3].

From a clinical and applied standpoint, this supports the notion of moving toward cycle-aware, but not rigidly “cycle-synced,” strategies: for most healthy, well-nourished women, modest phase-dependent adaptations—such as ensuring adequate energy and micronutrient intake in the mid-luteal phase, or considering the timing of high-intensity exercise relative to phases associated with lower subjective well-being—are likely to be sufficient, whereas in conditions such as PCOS, obesity, diabetes or functional hypothalamic amenorrhea, a more deliberate alignment of nutrition and training with endocrine and inflammatory profiles may help stabilize glycemic control, ovulatory function, and symptom burden [95,172].

At the same time, the evidence synthesized in this work underscores that menstrual cycle effects on nutrient metabolism likely arise from an integrated psychoneuroimmunoendocrine and microbiota network, rather than from isolated shifts in E2 and P4. Fluctuations in HPA axis activity, central insulin sensitivity, autonomic tone, gut microbial composition, intestinal permeability, and behavior (e.g., hedonic eating, sleep, spontaneous physical activity) co-evolve with cycle phase and may help explain why ostensibly similar hormonal profiles can yield divergent metabolic phenotypes across individuals or across cycles in the same woman [84,95,171,173].

For research design, this supports the need to systematically verify cycle phase with hormonal or ovulatory markers, explicitly report cycle characteristics, and—where feasible—stratify or adjust for phase, rather than excluding women or treating the menstrual cycle as statistical “noise.” It also highlights the need for multi-system study designs that incorporate endocrine, immune, microbiome, and behavioral readouts, and for sex-specific analyses that recognize that “female vs. male” metabolic differences are, in practice, comparisons between a dynamic infradian system and a more stable endocrine context [86,174].

Future research should build on this framework by prioritizing well-powered, longitudinal, and mechanistically integrated study designs that combine precise cycle phase verification with multi-omics approaches (e.g., metabolomics, transcriptomics, and microbiome profiling). Greater standardization of methodological approaches—including hormonal confirmation of cycle phase, harmonized exercise and nutritional protocols, and improved characterization of participant phenotypes—will be essential to reduce heterogeneity and improve comparability across studies. In addition, there is a need for more human-based mechanistic research to validate pathways that are currently inferred from preclinical or indirect evidence, particularly those related to mitochondrial function, substrate partitioning, and micronutrient metabolism. From an applied perspective, future work should also explore the feasibility and efficacy of individualized, cycle-aware nutritional and training strategies in both healthy and clinical populations, moving toward precision approaches that account for intra-individual variability across cycles.

From a translational perspective, advancing this field will require a shift toward integrating menstrual cycle physiology into both research frameworks and clinical practice. In science, this implies moving beyond treating the menstrual cycle as a confounder and instead incorporating it as a biologically meaningful variable in study design, data interpretation, and reporting standards. In clinical and applied settings, there is a need to transition from generalized, “one-size-fits-all” approaches toward more individualized, cycle-aware strategies that account for temporal metabolic variability. This includes the development of practical tools for cycle phase monitoring, the incorporation of endocrine-informed nutritional and exercise guidelines, and the recognition of menstrual health as a key component of metabolic health. Ultimately, bridging the gap between mechanistic research and real-world application will be essential to improve prevention, diagnosis, and intervention strategies in women’s metabolic health.

## Data Availability

No new data were created or analyzed in this study. Data sharing is not applicable to this article.

## Supplementary Materials

The following supporting information can be downloaded at: https://www.mdpi.com/article/10.3390/nu18071063/s1, Supplementary Table S1. Summary of menstrual cycle–related changes in macronutrient metabolism in eumenorrheic women. Supplementary Table S2. Summary of menstrual cycle–related changes in micronutrient metabolism and bioactive compounds in eumenorrheic women.

## Abbreviations

The following abbreviations are used in this manuscript:

ADIPO-IR	Adipose tissue insulin resistance index
ApoB	Apolipoprotein B
ATP	Adenosine triphosphate
AUC	Area under the curve
BCAA	Branched-chain amino acids
BMI	Body mass index
CGM	Continuous glucose monitoring
DHA	Docosahexaenoic acid
E2	Estradiol
EPA	Eicosapentaenoic acid
ER	Estrogen receptor
ERα	Estrogen receptor alpha
ERβ	Estrogen receptor beta
FSH	Follicle-stimulating hormone
GLP-1	Glucagon-like peptide-1
Hb	Hemoglobin
HDL-C	High-density lipoprotein cholesterol
HOMA-IR	Homeostatic model assessment of insulin resistance
HPO axis	Hypothalamic–pituitary–ovarian axis
iPTH	Intact parathyroid hormone
IVGTT	Intravenous glucose tolerance test
LDL-C	Low-density lipoprotein cholesterol
LH	Luteinizing hormone
LPC	Lysophosphatidylcholine
LPE	Lysophosphatidylethanolamine
MUFAs	Monounsaturated fatty acids
NK cells	Natural killer cells
OGTT	Oral glucose tolerance test
P4	Progesterone
PC	Phosphatidylcholine
PCOS	Polycystic ovary syndrome
PGC-1α	Peroxisome proliferator-activated receptor gamma coactivator-1 alpha
PYY	Peptide YY
PUFAs	Polyunsaturated fatty acids
Ra	Rate of appearance
Rd	Rate of disappearance
REE	Resting energy expenditure
ROS	Reactive oxygen species
SCFAs	Short-chain fatty acids
SF	Serum ferritin
SHBG	Sex hormone-binding globulin
sTfR	Soluble transferrin receptor
SERM	Selective estrogen receptor modulator
TC	Total cholesterol
TFA	Trans-fatty acids
TG	Triglycerides
T1D	Type 1 diabetes
VO_2_max	Maximal oxygen consumption
25(OH)D	25-hydroxyvitamin D
1,25(OH)_2_D	1,25-dihydroxyvitamin D
