# Supplementary material for: An Overview of the Impact of the Menstrual Cycle on Nutrient Metabolism: An Integrative Perspective
Source: Nutrients. 2026 Mar 26;18(7):1063. doi: 10.3390/nu18071063 (PMC13074570; doi:10.3390/nu18071063)
Supplement: Supplementary file 1 [file nutrients-18-01063-s001.zip › nutrients-4217462-supplementary.pdf]

Supplementary Table S1. Summary of menstrual cycle–related changes in macronutrient metabolism in eumenorrheic women.

Macronutrient	Main menstrual cycle pattern	Magnitude / consistency	Main proposed mechanisms	Key references
**Glucose tolerance / insulin sensitivity**	Glucose tolerance and insulin sensitivity are generally more favorable in the follicular phase and slightly reduced in the luteal phase	Modest but relatively consistent in contemporary studies; substantial interindividual variability	E2 tends to enhance insulin sensitivity, whereas P4 partially antagonizes insulin action; effects are further modulated by BMI, fitness, diet, sleep, and behavioral factors	[4,95–97]
**Fasting glucose, insulin and HOMA-IR**	Fasting insulin and HOMA-IR tend to peak in the luteal phase; fasting glucose changes are smaller and less consistent	Small, but supported by large population datasets	Relative luteal insulin resistance, with amplification in women with higher BMI and/or lower fitness	[96,101]
**Free-living glycemia / diabetes context**	In healthy women, mean glycemia is slightly higher in the luteal phase; in T1D/T2D, this pattern is often more pronounced, with higher insulin requirements luteally	Small in healthy women; clinically more relevant in diabetes	Reduced insulin sensitivity during the luteal phase; incomplete compensation by insulin secretion	[98–100]
**Carbohydrate use during exercise**	The luteal phase is often associated with glycogen sparing, reduced glucose turnover, and lower hepatic glucose output during submaximal exercise	Modest and protocol-dependent	Greater E2-related lipid oxidation reduces carbohydrate reliance; P4 may modulate this effect	[103–107]
**Effect of carbohydrate availability**	Adequate carbohydrate intake or supplementation tends to attenuate or override phase-related differences in glycogen use and performance	Fairly consistent practical observation	Exogenous carbohydrate availability diminishes the relevance of endogenous hormonal modulation of substrate partitioning	[104,109]
**Energy intake and carbohydrate-related eating behavior**	Energy intake is generally higher in the luteal phase, often with a tendency toward more simple sugars / energy-dense foods and relatively less complex carbohydrate	Moderate, though not universal across studies	Appetite effects of P4 in the presence of E2; modulation by leptin, ghrelin, PYY, and central insulin signaling; partly compensatory to higher energy expenditure	[84,110–114]

Macronutrient	Main menstrual cycle pattern	Magnitude / consistency	Main proposed mechanisms	Key references
**Plasma lipids: TC and LDL-C**	Total cholesterol (TC) and LDL-C tend to be higher in the follicular phase and lower in the luteal phase	Modest but relatively consistent	E2 reduces hepatic lipase activity and upregulates LDL receptor pathways; P4 may exert counter-regulatory effects	[124,125,131–133]
**Plasma lipids: HDL-C and TG**	HDL-C tends to peak in the late follicular/periovulatory phase; TG findings are less consistent, though some studies report higher follicular values	Small to modest, with notable heterogeneity	Acute E2-related HDL changes; interactions with insulin sensitivity, adipokines, and lifestyle	[83,124,125,96]
**Lipid-related cardiovascular ratios**	TC/HDL-C, LDL-C/HDL-C, and TG/HDL-C are generally more favorable in the luteal phase	Small absolute differences	Integrated effects of ovarian steroids, insulin sensitivity, and adipose tissue metabolism	[125,126,96,131]
**Lipidomic patterns / fat oxidation**	Several phospholipids decrease in the luteal phase, while acylcarnitines may rise periovulatorily; fat oxidation during exercise is often higher in the luteal phase, especially at submaximal intensity	Modest, less consistently reproduced than classical lipids	Hormonal modulation of membrane lipid turnover and fatty acid transport/β-oxidation	[3,8,107,126,134]
**Protein and amino acid profile**	The luteal phase shows a slightly more catabolic profile, with lower circulating concentrations of several amino acids, including BCAAs	Subtle but recurrent across metabolomic/tracer studies	P4 may increase amino acid utilization for reproductive tissues; E2 may exert protein-sparing effects	[3,96,138]
**Protein turnover / synthesis**	Whole-body and myofibrillar protein synthesis appear largely unchanged across phases, despite lower plasma amino acids luteally	Relatively consistent in controlled tracer studies	Lower circulating amino acids likely reflect increased flux, oxidation, or redistribution rather than reduced anabolic capacity	[139,140]
**Amino acid oxidation / requirements**	Leucine oxidation and flux, and possibly lysine requirements, appear slightly higher in the luteal phase	Modest, based on relatively few studies	Greater amino acid catabolism and contribution to energy metabolism in the luteal phase	[108,141]

Abbreviations: BCAA, branched-chain amino acids; E2, estradiol; HDL-C, high-density lipoprotein cholesterol; HOMA-IR, homeostatic model assessment of insulin resistance; LDL-C, low-density lipoprotein cholesterol; P4, progesterone; TC, total cholesterol; TG, triglycerides; T1D, type 1 diabetes. **Note**: Reported changes are generally modest in magnitude and strongly influenced by study design, phase verification, metabolic status, physical activity, and dietary intake.

Supplementary Table S2. Summary of menstrual cycle–related changes in micronutrient metabolism and bioactive compounds in eumenorrheic women

Micronutrient / bioactive compound	Main menstrual cycle pattern	Magnitude / consistency	Main proposed mechanisms	Key references
**One-carbon metabolism (folate / homocysteine)**	Serum folate tends to be lowest during menses and higher in the luteal phase, whereas homocysteine is generally higher in the follicular/periovulatory phase and lower in the luteal phase	Most consistent vitamin-related pattern across the cycle, although still modest in magnitude	Likely reflects hormonally patterned shifts in methyl-group demand, oxidative stress, inflammatory tone, and luteal function; more relevant as a biomarker pattern than evidence of changing dietary requirements	[143,46]
**B-vitamins (B2, B6, B12 intake)**	No clear robust within-cycle biomarker oscillation established beyond one-carbon metabolism; adequate intake appears to support cycle metabolic resilience	Limited to modest; not supportive of phase-specific dosing	B-vitamins appear to function more as metabolic capacity factors than as direct cycle regulators in well-nourished women	[144]
**Vitamin D status / metabolism**	25(OH)D usually remains stable across phases; 1,25$(OH)_2$D and iPTH may show small and inconsistent fluctuations	Modest and inconsistent for active metabolites; stable for 25(OH)D in most studies	Functional vitamin D signaling may still interact with reproductive hormones despite stable 25(OH)D; current evidence does not support altered intra-cycle requirements in vitamin D–replete women	[145,146]
**Vitamin D and cycle characteristics**	Lower vitamin D status is associated with longer cycles, longer follicular phases, and possible shorter luteal phases	Observational but clinically relevant, especially in deficiency/insufficiency	Suggests that adequate vitamin D status may support ovulatory competence and luteal adequacy, although this reflects baseline status more than phase-specific change	[147]
**Antioxidant vitamins (C, A, E)**	Available longitudinal studies show minimal or inconsistent phase-related changes	Weak / sparse evidence; generally smaller than one-carbon or iron-related variation	Variations appear to remain within normal biological variability; no convincing evidence for robust follicular–luteal cycling	[150,151]

Micronutrient / bioactive compound	Main menstrual cycle pattern	Magnitude / consistency	Main proposed mechanisms	Key references
**Iron biomarkers**	Hemoglobin, ferritin and transferrin saturation generally nadir during menses and recover/peak later, whereas sTfR rises transiently during menses; hepcidin is lowest during menses and higher in the early luteal phase	Strongest and most physiologically robust micronutrient-related cycle pattern	Primarily driven by menstrual blood loss, erythropoietic response, and hepcidin–iron regulation; biomarker timing is highly phase-sensitive	[152-154]
**Iron deficiency risk modifiers**	Heavy menstrual bleeding, low iron intake, obesity/inflammation amplify cycle-related iron fluctuations	Clinically relevant in susceptible subgroups	Supports phase-aware biomarker interpretation and targeted screening rather than universal phase-specific supplementation	[154,155]
**Zinc**	Often higher around mid-follicular / ovulatory phases and lower during menses or mid-luteal phase, depending on cohort	Modest and somewhat inconsistent	May reflect hormonal regulation of transport and tissue demand; evidence supports measurable variability but not clear clinical depletion in well-nourished women	[155,157,160]
**Magnesium**	Small decline from early follicular to mid-luteal phase has been reported; magnesium deficiency may be more frequent luteally in some cohorts	Modest, based on relatively few studies	Could relate to renal handling, tissue demand, and greater luteal metabolic load; evidence insufficient for routine phase-specific supplementation	[155,157,161]
**Selenium / copper / calcium**	Selenium may fluctuate modestly across phases; copper appears relatively stable in circulation; calcium relevance is more linked to symptom modulation than cyclical biomarker change	Limited / heterogeneous	Selenium may contribute to follicular maturation and redox control; calcium adequacy may help premenstrual symptoms; tissue-level status may be more informative than serum levels alone	[158,159]

Micronutrient / bioactive compound	Main menstrual cycle pattern	Magnitude / consistency	Main proposed mechanisms	Key references
**Overall trace mineral handling**	Trace mineral changes are generally smaller and less consistent than iron changes	Modest, heterogeneous, and often observational	Functional significance remains uncertain in healthy women; adequacy and high-risk subgroup screening are more evidence-based than phase-targeted dosing	[155,157,160–162
**Phytoestrogens (isoflavones / lignans)**	No clear endogenous cycle fluctuation; habitual intake is associated with modest, context-dependent effects on menstrual regularity and reproductive hormones	Small to moderate observational associations	Likely act via weak SERM-like effects, especially through ERβ, and may modestly affect SHBG and androgens without overt disruption of ovulatory hormones	[163–166]
**Polyphenols / carotenoids**	No robust phase-specific fluctuation established; functional relevance may be greater in higher oxidative-stress contexts, potentially including the luteal phase	Limited and indirect evidence	Likely act through antioxidant and anti-inflammatory effects rather than through direct cycle-phase regulation; overall diet quality appears more relevant than phase-specific use	[167]
**Gut microbiota–derived bioactive metabolism**	Interindividual variability in microbial metabolism likely modifies responses to phytoestrogens and polyphenols more than the menstrual cycle itself	Preliminary / mechanistically plausible	Microbial conversion (e.g., equol, urolithins) may determine bioactivity, but evidence does not support defined phase-specific dosing strategies	[65,166,168,169,170]

Abbreviations: $1,25(OH)_2D$, 1,25-dihydroxyvitamin D; 25(OH)D, 25-hydroxyvitamin D; ERβ, estrogen receptor beta; FSH, follicle-stimulating hormone; iPTH, intact parathyroid hormone; sTfR, soluble transferrin receptor; SERM, selective estrogen receptor modulator; SHBG, sex hormone-binding globulin.

Note: Most reported changes reflect fluctuations in circulating biomarkers rather than demonstrated changes in whole-body stores or dietary requirements. Iron-related indices show the most robust cyclical variation; for most other micronutrients and bioactive compounds, evidence supports adequacy-based rather than phase-specific nutritional strategies.
